# Cancer radioresistance is characterized by a differential lipid droplet content along the cell cycle

**DOI:** 10.1186/s13008-024-00116-y

**Published:** 2024-04-20

**Authors:** Francesca Pagliari, Jeannette Jansen, Jan Knoll, Rachel Hanley, Joao Seco, Luca Tirinato

**Affiliations:** 1 Division of Biomedical Physics in Radiation Oncology, German Cancer Research Center (DKFZ), 69120 Heidelberg, Germany; 2 Department of Physics and Astronomy, Heidelberg University, Im Neuenheimer Feld, 69120 Heidelberg, Germany; 3 Department of Medical and Surgical Science, University Magna Graecia, 88100 Catanzaro, Italy

**Keywords:** Lipid droplets, Radioresistance, Cancer metabolism, Cell cycle, Perilipins

## Abstract

**Background:**

Cancer radiation treatments have seen substantial advancements, yet the biomolecular mechanisms underlying cancer cell radioresistance continue to elude full understanding. The effectiveness of radiation on cancer is hindered by various factors, such as oxygen concentrations within tumors, cells’ ability to repair DNA damage and metabolic changes. Moreover, the initial and radiation-induced cell cycle profiles can significantly influence radiotherapy responses as radiation sensitivity fluctuates across different cell cycle stages. Given this evidence and our prior studies establishing a correlation between cancer radiation resistance and an increased number of cytoplasmic Lipid Droplets (LDs), we investigated if LD accumulation was modulated along the cell cycle and if this correlated with differential radioresistance in lung and bladder cell lines.

**Results:**

Our findings identified the S phase as the most radioresistant cell cycle phase being characterized by an increase in LDs. Analysis of the expression of perilipin genes (a family of proteins involved in the LD structure and functions) throughout the cell cycle also uncovered a unique gene cell cycle pattern.

**Conclusions:**

In summary, although these results require further molecular studies about the mechanisms of radioresistance, the findings presented here are the first evidence that LD accumulation could participate in cancer cells’ ability to better survive X-Ray radiation when cells are in the S phase. LDs can represent new players in the radioresistance processes associated with cancer metabolism. This could open new therapeutic avenues in which the use of LD-interfering drugs might enhance cancer sensitivity to radiation.

## Background

Although cancer therapies are rapidly evolving and significantly improving treatment options and outcomes, much still needs to be understood, particularly the biomolecular mechanisms that drive cancer cell resistance. Radiotherapy remains one of the most widely used cancer treatment approaches, alongside surgery, chemotherapy, and the more recent immunotherapy. Ionizing radiation is employed to treat nearly all types of solid tumors, though radiosensitivity varies among tumor types. For instance, some neoplasms, like lymphomas, respond very well to radiotherapy even at low doses [1], while others exhibit high radiation resistance, such as gliomas [2]. Moreover, cancer staging and individual patient characteristics significantly contribute to diverse radioresponses even within the same cancer type. These “therapeutic failures” arise from the intricate biological complexity of the tumor mass. Biological factors driving or associated with radioresistance have been studied for several decades. Among them, the varying oxygen concentrations within the tumor mass and the cellular DNA damage repair capacity are some of the most prominent. These factors contribute to an intrinsic heterogeneity among cancer cells, resulting in differing sensitivities to radiation therapy [3]. In general, hypoxic non-cycling cells as well as quiescent cancer stem cells are more resistant to radiation than normoxic cycling cells [3–5]. Indeed, during radiation treatment, the proliferation rate of more radiosensitive cells decreases due to cell cycle arrest, which is prompted by the activation of the DNA Damage Response (DDR) machinery. This subsequently induces DNA damage repair or cell death mechanisms [6, 7]. However, irradiated cancer cells (both hypoxic and normoxic) can develop evasion strategies to cell death, and even metastatic processes can be triggered after radiation treatments [8].

Therefore, both the initial cell cycle profiles and the radiation-induced cell cycle redistribution considerably influence the responses to radiotherapy. Deregulation of cell cycle profiles is one of the hallmarks of cancer [9], and it has been shown that radiation sensitivity varies along the different cell cycle phases. In fact, cells in G0, early G1, and especially S phases are more resistant to ionizing radiation, while, in G2 and M phases, cells are more sensitive [7].

Lipid Droplets (LDs) are cytoplasmic organelles rich in lipids currently well recognized as active and dynamic platforms with several functions in cancer development and progression, and they have been associated with poor prognosis in patients [10]. Moreover, in an interesting paper published by Cruz et al. [11], it was demonstrated that LD accumulation was regulated along the cell cycle in mouse fibroblasts.

Key regulators of LD dynamics and metabolism are a group of LD-membrane-associated proteins called Perilipins (PLIN). In mammals, the PLIN family consists of five members (from PLIN1 to PLIN5) with specific tissue distribution and functional roles, although some overlaps exist [10, 12]. In particular, PLIN1 is primarily known for its role in regulating lipid metabolism in adipocytes. There is also some evidence suggesting a potential link between PLIN1 and tumor progression, chemoresistance, and poor prognosis in certain cancer types, such as breast cancer. However, conflicting data have been reported and PLIN1-associated better prognosis has been also observed [13]. PLIN2 is widely expressed in various cell types and is involved in lipid storage, LD formation, and turnover [10, 14]. PLIN2 expression levels have been investigated as a potential diagnostic and prognostic marker in cancer. Elevated PLIN2 expression has been in fact correlated with advanced tumor stage, lymph node metastasis, and reduced overall survival in some cancer types [15].

PLIN3 is associated with lipid trafficking between LDs and other organelles. Instead, PLIN4 is involved in lipid metabolism mainly in steroidogenic tissues, while PLIN5 primarily plays a role in regulating lipid oxidation and mitochondrial function [12, 14]. However, PLIN4 and PLIN5 roles in cancer are relatively underexplored. Some studies have shown upregulated expression of both PLINs in certain cancer types, such as in some breast cancer tissues and hepatocellular carcinoma [16]. On the contrary, in other studies opposite results have also been shown [17].

Upregulated lipogenesis and increased LD content in tumor tissues have been linked to a cancer stem cell (CSC) phenotype in several types of tumors such as colon [18], breast [19], and glioblastoma [20]. Our group has shown that LDs are a signature of cancer radioresistance in various cancer cell lines (prostate, lung, breast, bladder, and neuroglioma) and that their intracellular abundance is tightly correlated to iron metabolism, especially to the ferritin heavy chain 1 expression [21]. Interestingly, we have also demonstrated that LD inhibition, through chemical DGAT2 downregulation, increased DNA damage after X-ray irradiation and reduced the expression of some CSC markers in breast cancer cells, which resulted more radiosensitive [22].

All this evidence suggests a significant role for LDs in cancer, particularly in the context of radioresistance, a research field which is currently under investigated.

The present work aimed at analyzing the LD content throughout the cell cycle in lung (H460) and bladder (T24) cancer cell lines. The goal was to evaluate a potential correlation between the cell cycle phase distribution and cell radiosensitivity in two cell lines from human lung and bladder cancer.

## Results and discussions

### Cancer cells exhibit particularly abundant lipid droplet content during the S phase

**Fig. 1:**
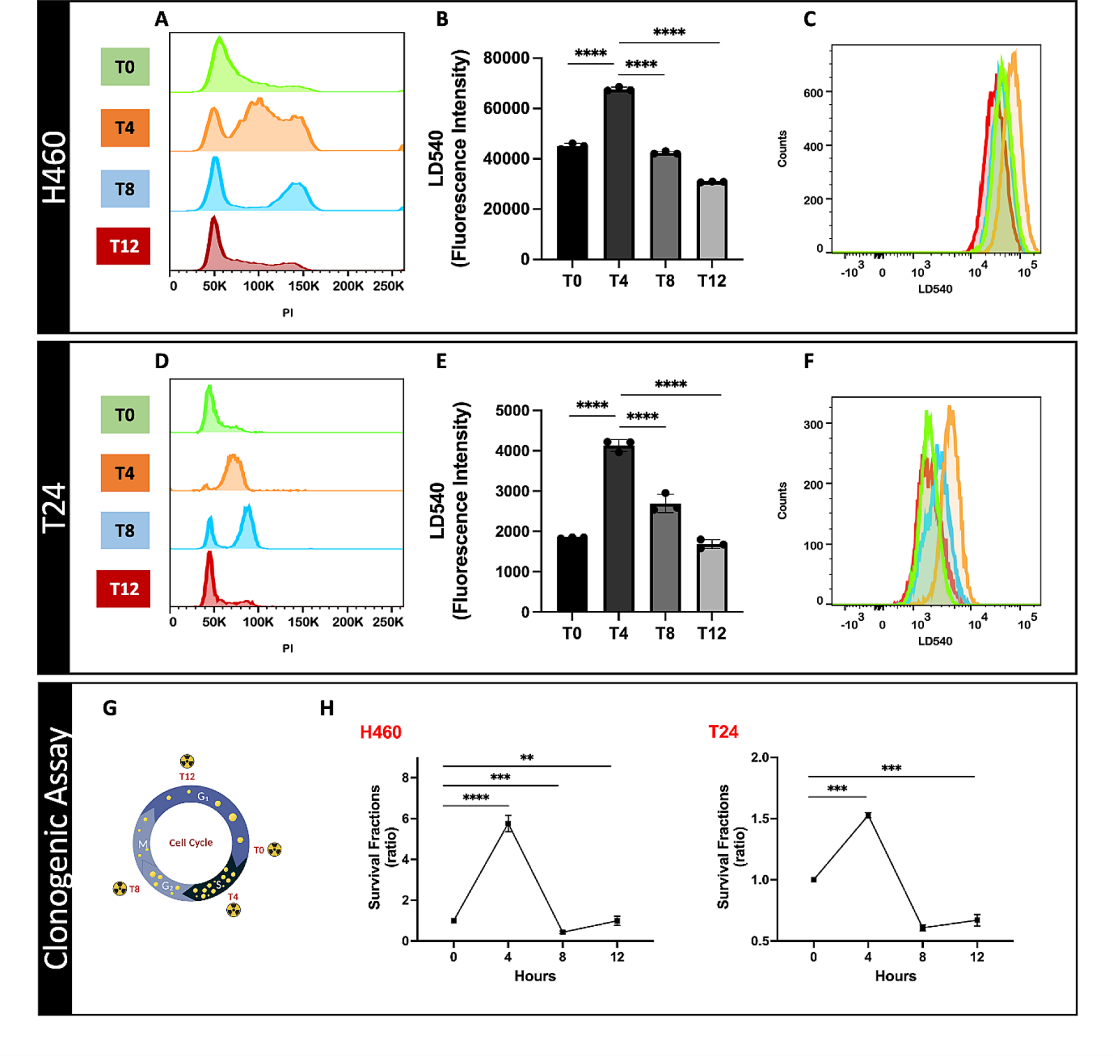
Upper panel: (A-C) Lipid Droplet distribution and quantification along the cell cycle in human lung cells (H460). Middle panel: (D-F) Lipid Droplet distribution and quantification along the cell cycle in human bladder cancer cells (T24). Lower panel: (G) schematic representation of LD content in cells along the cell cycle phases and its relationship with cancer radioresistance; (H) Survival curves of synchronized H460 and T24 cells at different time-points irradiated with 6 Gy X-rays as assessed by clonogenic assays. The data represent the mean of three biological replicates ± SD. The clonogenic assay values were normalized to the T0 value, representing the late G1 phase. T0 was set as 1, and the subsequent time points (T4, T8, and T12) were compared to it. (* ≤ 0.05; ** ≤ 0.01; *** ≤ 0.001 and ****≤ 0.0001)

To analyze the cellular LD amount during the cell cycle progression, H460 and T24 cells were first synchronized using an arrest-release technique. For this purpose, HU, which reversibly inhibits DNA replication and therefore induces cell cycle arrest at the G1-S phase border, was used. Afterward, cells were released by removing HU and adding fresh medium thus allowing to monitoring cell cycle progression at different time-points (T0, T4, T8, and T12 hours after release). FACS analysis on propidium iodide-stained samples confirmed cell synchronization. Figure 1A and D illustrate the synchronization of each sample at the respective time-points, showing an enrichment for cells in late G1 (T0), S phase (T4), G2-M phases (T8), and the re-entry into the G0-G1 phase (T12) (Table 1).

**Table 1:** % of cells in each phase of the cell cycle after synchronization with HU

H460		T24	
T0	*G0-G1*: 75.3%, *S*: 15.3%, *G2/M*: 7.89%	T0	*G0-G1*: 79.4%, *S*: 20.2%, *G2/M*: 3.1%
T4	*G0-G1*: 19.4%, *S*: 53.5%, *G2/M*: 24.8%	T4	*G0-G1*: 7.3%, *S*: 70.7%, *G2/M*: 22.3%
T8	*G0-G1*: 43%, *S*: 7.45%, *G2/M*: 46.2%	T8	*G0-G1*: 27.2%, *S*: 10.8%, *G2/M*: 62.3%
T12	*G0-G1*: 64%, *S*: 19.2%, *G2/M*: 15.2%	T12	*G0-G1*: 70.7%, *S*: 16.5%, *G2/M*: 13.7%

The cycle duration from late G1 to a new G0-G1 was approximately 12 hours (h). In general, synchronization was maintained in the cells for at least 12 h. Importantly, previous studies indicated that an 18 h HU treatment in a complete medium did not reduce cell viability (data not shown), demonstrating that the concentration and incubation time used in these studies were not sufficient to induce cytotoxic effects.

After establishing the cell cycle synchronization protocol, the same experimental approach was employed to assess LD regulation throughout the different cell cycle phases. Cells were stained with the lipophilic dye LD540 at specified time points and immediately analyzed via flow cytometry to quantify the LD content. An increase in LDs was observed at T4 (corresponding to the S phase) in both cell lines (Fig. 1B, C, E, and F), which was followed by a decrease during the G2-M and G0-G1 phases (T8 and T12, respectively). These findings illustrate that LD accumulation in cancer cells is regulated during cell cycle progression and the observed cytoplasmic LD increase in the S phase was consistent with the observations by Cruz A. et al. [11]. These authors reported that LDs typically accumulated during the S phase in non-transformed murine fibroblasts and a rat epithelial cell line. Moreover, although LD accumulation was also observed in transformed murine fibroblasts, no evidence of LD regulation throughout the cell cycle was reported. Therefore, to our knowledge, this is the first study demonstrating a cell-cycle dependency of LD content in cancer cells, specifically highlighting an increase during the S phase.

Previous research has shown that lipid metabolism is closely coordinated with the cell cycle [23], especially during the S phase, where a significant accumulation of phospholipids is observed [24]. This increased lipid biosynthesis aids in sustaining rapid growth in cancer cells and ensures an adequate supply of cellular lipid components in preparation for cell division.

However, the differential enrichment of LDs throughout the cell cycle, particularly the accumulation during the S phase, may also serve additional purposes. LDs are organelles rich in cholesterol and triacylglycerols, both of which are key regulators in cell signaling. For instance, studies have shown that elevated cholesterol levels can enhance radioresistance in colorectal cancer cells [25], induce epithelial-to-mesenchymal transition (EMT) in prostate cancer [26], and contribute to the pathophysiology of breast [27] and bladder cancers [28].

### Cancer cell radioresistance correlates with lipid droplet content and perilipin expression is modulated along the cell cycle

Building upon our prior research, which showed an accumulation of LDs in radioresistant cancer cells [21, 22], we decided to further investigate the potential correlation between radioresistance and LD content at various stages of the cancer cell cycle.

It is well established that cancer cells exhibit varying levels of radiosensitivity throughout the cell cycle, with cells in the S phase showing the highest radioresistance [7]. To this purpose, synchronized cells at different cell cycle phases were irradiated with 6 Gy X-ray (Fig. 1G). All irradiated samples were cultured for 10 days, after which surviving colonies were counted. Intriguingly, both cell lines showed the greatest clonogenic potential when irradiated during the S phase (T4). In the other phases instead, the clonogenic capacity decreased relative to the S phase, and this decrease was cell-type specific (Fig. 1H).

These results not only reinforce previous findings that cells in the S phase are more radioresistant, but they also strengthen this knowledge by showing concurrent accumulation of cytoplasmic LDs during this phase. Therefore, this evidence suggests a potential role of these organelles in participating in cancer cell radioresistance. However, it should be noted that our findings currently show a correlation between LD increase during the S phase and radioresistance without establishing a causative relationship. Future studies are necessary to elucidate the mechanistic underpinnings of this association and to explore potential therapeutic implications.

The PLIN family, comprising PLIN 1, 2, 3, 4, and 5, is a class of proteins associated with LDs and involved in their biogenesis, structure, and function [10, 12]. Furthermore, altered PLIN expression has been associated with various types of cancer and has been proposed as a potential prognostic biomarker [10, 15]. Consequently, we were interested in whether the different PLIN genes exhibited differential expression throughout the various cell cycle phases, and how the expression of each PLIN mRNA changed over time.

**Fig. 2:**
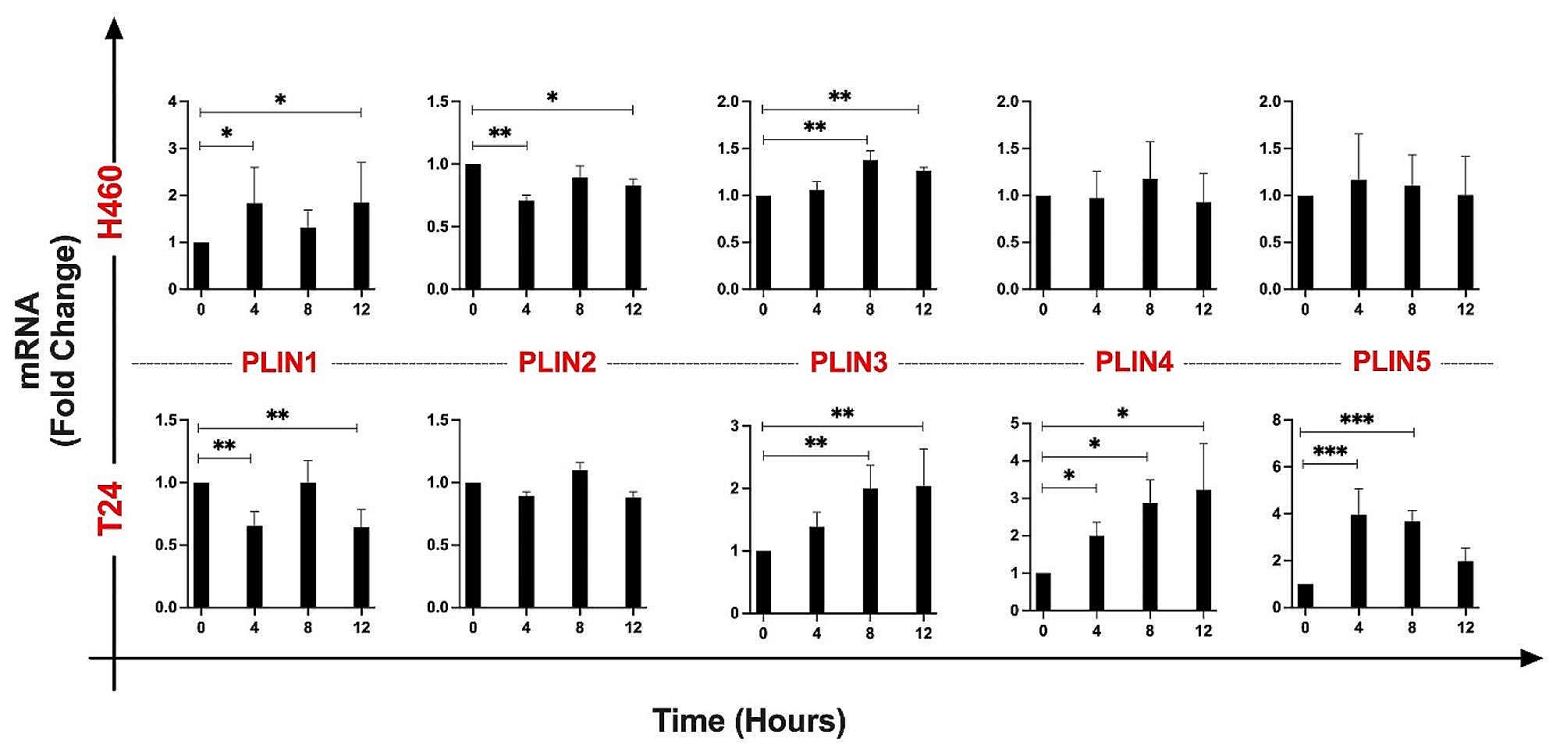
PLIN gene expression in synchronized H460 and T24 cell lines. Specifically, T0 represents the late G1 phase, T4 corresponds to the S phase, T8 to the G2-M phase, and T12 to the re-entry into G0-G1. The data represent the mean of three biological replicates ± SD. Each time-point was normalized and statistically compared to the respective T0 (* ≤ 0.05; ** ≤ 0.01; *** ≤ 0.001 and ****≤ 0.0001)

To explore these questions, RT-PCR on RNAs extracted from cells that were synchronized at different phases of the cell cycle was performed (Fig. 2).

H460 cells showed an increase of PLIN1 at T4 and T12, a decrease of PLIN2 at T4, and an increase at T12 compared to the T0. PLIN3 mRNA instead increased at T8 and T12, while no significant changes were observed for both PLIN4 and PLIN5.

T24 cells were characterized by a major modulation of almost all the PLIN genes, with the exception of PLIN2. Specifically and conversely to H460 cells, PLIN1 was downregulated at T4 and T12. Instead, PLIN3 mRNA showed a similar behavior as in H460 cells with increased expression at T8 and T12. As far as PLIN4 expression is concerned, a significant time-dependent increase was observed, while PLIN5 mRNA levels were upregulated at T4 and T8.

That elevated expression of PLIN1 is linked with improved prognosis in some cancers [13], in this study we may hypothesize that the upregulation of PLIN1 at T4 in H460 cells, when compared to T24 cells, might be one of several potential factors contributing to their differential radiosensitivity. When examining the survival curves derived from the clonogenic assays, it was evident that H460 cells exhibited an overall lower degree of radioresistance in comparison to T24 cells. Notably, T24 cells, characterized by high malignancy and invasiveness, showed a downregulation of PLIN1 expression during the S phase. However, at the moment, this hypothesis remains to be investigated. Additionally, it is yet to be determined whether PLIN1 protein localization on LD organelles is indeed diminished in H460 cells despite the observed increase in LD count during the S phase. Further research in this direction can provide a better understanding of the observed differences in PLIN1 expression and it can help to fully elucidate its significance.

Additionally, high PLIN2 expression has been linked to poor prognosis in lung cancer. Our results showed a PLIN 2 downregulation in H460 cells at T4, further suggesting a potential link with their general radiosensitivity. In T24 cells, PLIN4, which is involved in LD stability, and PLIN5, likely playing a protective role in fatty acid overload and oxidative stress [29], were predominantly upregulated in the S phase. It has been shown that PLIN4 mRNA and protein levels were highly and exclusively expressed in chemoresistant triple-negative breast cancer [16], whereas, in another study, PLIN4 expression was a favorable factor for overall survival in bladder cancer patients [17]. However, it should be noted that none of these studies evaluated the expression of PLINs throughout the cell cycle, but only as a general feature of the analyzed samples, which might not accurately reflect a time-dependent expression pattern.

Regarding the role of LDs in radioresistance, the understanding is largely unknown. It is established that reactive oxygen species (ROS) levels increase as cells progress from G1 to S phase in various in vitro cell models [30, 31]. In addition, radiotherapy works by inducing DNA damage either directly or through ROS production, which subsequently leads to cell cycle arrest. Research has shown that an increase in ROS can be associated with an increase in LD amount, which could act as an antioxidant strategy [21, 32, 33]. In this context, the accumulation of lipids in LDs could serve as a defense against excessive IR-induced oxidative stress, which can cause lipid peroxidation resulting in the production of unstable lipid radicals, such as lipid hydroperoxide (LOOH), and more stable, yet reactive and toxic, compounds, such as aldehydes. These latter compounds have been implicated in the pathogenesis of many diseases [34, 35] and have been proven to be genotoxic by interacting with the DNA molecule [36]. Such an interaction might amplify the damaging effects of radiation treatments on membranes and newly synthesized DNA molecules during the S phase.

Consequently, we could hypothesize that in a heterogeneous cell population subjected to X-ray irradiation, cells in the S phase might be more resistant to IR-induced damage compared to other cell subsets partly due to the accumulation of LDs that protect S phase cells from lipotoxicity.

## Conclusions

The role of the cell cycle in cancer radioresistance has been a subject of investigation for decades. Findings from this research have been used and implemented in clinical protocols, primarily through the use of fractionation schemes that allow surviving cancer cells to redistribute across the cell cycle and initiate DNA repair processes. Despite these advances, a deeper understanding of the underlying mechanisms and the identification of potential targets represent critical steps to enhance the sensitivity of cancer cells to treatments. Although there is still much to be understood at the cellular and molecular level, the increased LDs in the S phase observed in this study for the first time hints at the potential involvement of these organelles in cancer radioresistance. This could open up new scenarios for developing new radiosensitizers, thereby shaping future oncological strategies.

## Materials and methods

### Cell culture

Human non-small lung cancer cells, H460 (ATCC), were cultured in RPMI 1640 medium (Thermo Fisher Scientific), while the urinary bladder carcinoma cell line, T24 (ATCC), was grown in McCoy’s medium (Thermo Fisher Scientific). All media were completed with the addition of 10% Fetal Bovine Serum (FBS) and 1% penicillin-streptomycin (complete medium) (both from Thermo Fisher Scientific). Both cell lines were maintained at 37 °C in a humidified atmosphere with 5% CO_2_, following the recommendations provided by ATCC.

### Cell cycle synchronization

To initially generate a homogeneously quiescent cell population and slow down cell growth in the G0-G1 phase, both cell lines (2.5 × 10^6^ cells) were seeded in a T175 flask (Greiner Bio-one). T24 cells were grown in 2% FBS for 24 h, while H460 cells were grown at 0% FBS for 48 h. After this period, both cell lines were washed thrice with 1X D-PBS (Sigma Aldrich) and cultured in their complete media containing 0.5 mM hydroxyurea (HU) (Sigma Aldrich) for another 18 h in order to synchronize cells in the late G1-S phase.

Afterwards, the HU was removed by washing the synchronized cells three times with 1X D-PBS and cells were then released into the cell cycle by adding the appropriate complete medium. The samples were collected at four-hour intervals, starting immediately after HU removal (0-, 4-, 8-, and 12-hrs post-release) to cover all cell cycle phases. Cells and cell pellets were harvested for further analysis as described below.

### Cell cycle analysis

At each time point, 1 × 10^6^ cells were collected and fixed by slowly adding 70% ice-cold ethanol while gently vortexing. All samples were sealed and stored at + 4 °C overnight. The following day, cell samples were pelleted, washed with 1X D-PBS, and treated with 100 units/mL of Ribonuclease A (Sigma Aldrich) for 30 min (min). Subsequently, the samples were washed and incubated with a staining solution containing 20 µg/mL Propidium Iodide (PI) (Thermo Fisher Scientific) in 1X PBS. The resulting DNA content was evaluated using a FACSCanto II flow cytometer (BD Bioscience) equipped with a blue laser (488 nm) and analyzed with FlowJo software 10 (Tree Star Inc.). Cells were considered synchronized when at least 70% of them accumulated in the late G1 phase.

### Lipid droplet staining

The LD content of all synchronized cells was analyzed using LD540 staining, as previously reported [11]. In brief, 4 × 10^5^ cells at various time-points along the cell cycle were harvested, washed twice with 1X D-PBS, and then stained with 0.1 µg/mL of the lipophilic dye LD540 (Enamine Ltd) in 1X-DPBS for 10 min. Cells were subsequently washed three times with 1X-DPBS. All samples were then analyzed using the FACSCanto II by exciting with a 488 nm laser and collecting the fluorescence emission at 530/30 nm.

### Clonogenic assays

T24 and H460 cells, synchronized in four distinct cell cycle phases (late G1, S, G2-M, and G0-G1) as described above, were seeded in T175 flasks at densities of 3.5 × 10^5^ and 1.0 × 10^6^ cells, respectively, six hours prior to irradiation to allow them to attach to the plastic surfaces. Subsequently, all samples were placed inside a MultiRad225/26 irradiator (Faxitron Biotics) (200 kV X-rays; 17.8 mA; 0.5 mm Cu-filter; 2.151 Gy/min) at a distance from the source of 37 cm, with a field size of 20 cm. Cells in T175 flasks were irradiated with 6 Gy X-rays. Soon after, the medium was replaced with fresh complete medium and the cells were cultured for ten days in a standard cell incubator. Cell survival was assessed using a standard colony-forming assay.

Upon completion of the incubation period, colonies were washed with 1X PBS, fixed in 100% ethanol, and stained with a 0.05% crystal violet solution. Only colonies comprising more than 50 cells were counted. The surviving fraction (SF) at a dose D (in Gy) was calculated after correction for the plating efficiency (PE) of control cells, as follows:

$${\rm{SF }}({\rm{D}})\, = {\rm{ Colonies counted }}/({\rm{Cells seeded }} \times \,{\rm{PE}}/100)$$

SF data were fitted to the Linear-Quadratic model [37].

### RNA extraction and semi-quantitative reverse transcriptase polymerase chain reaction (RT-qPCR)

For every time-point during the synchronization, cells were harvested, and total RNA was extracted from 10^6^ cells using the High Pure RNA isolation kit (Roche) according to the manufacturer’s instructions. Genomic DNA was digested with DNase I and the RNA amount and quality were then checked spectroscopically using a NanoDropND-1000 (NanoDrop Technologies). 1 µg total RNA per sample was reverse-transcribed using an RT2 First Strand Kit (Qiagen), according to the manufacturer’s instructions, in a StepOnePlus Real-time PCR system (Thermo Fisher Scientific). The synthesized cDNAs (20 ng) were amplified in 15 µL of a reaction mixture consisting of nuclease-free water, 20 pmol of each primer pair, and 7.5 µL of Power SYBR Green PCR Master Mix (Thermo Fisher Scientific). The amplification conditions were as follows: 95 °C for 10 min (1 cycle), 95 °C for 15 s, and 60 °C for 1 min (40 cycles). The primer sequences can be found in Table 2. GAPDH was used as internal control and all relative gene expressions were normalized to it.

**Table 2:** Nucleotide sequence list of the primer couples used for RT-qPCR.

Gene Name	Primer Forward 5’→3’	Primer Reverse 5’$\to$3’
PLIN1	CATTGAGAAGGTGGTGGAGT	GAGAGGGTGTTGGTCAGAG
PLIN2/ADRP	ACAGGGGTGATGGACAAGAC	ATCATCCGACTCCCCAAGAC
PLIN3/TIP47	CACCATGTTCCGGGACATTG	GCACCTGGTCCTTCACATTG
PLIN4	GTTCCAGGACCACAGACA	CCTACACTGAGCACATCC
PLIN5/ OXPAT	GATCACTTCCTGCCCATGAC	GCTGTCTCCTCTGATCCTCC
GAPDH	GCATCCTGGGCTACACTGAG	AAAGTGGTCGTTGAGGGCA

### Data analysis

Statistical analysis was carried out using GraphPad Prism software (Version 10) and the data were graphed using the same software. Three independent experiments have been performed. The data are displayed as average values ± standard deviation (SD) for each sample compared to the control. The T-test and one-way ANOVA were used to determine statistical significance and a p-value of 0.05 was established as the cut-off.

## Data Availability

All data generated not included in this study are available and will be shared upon request to the corresponding authors.
